# Role of the Extracellular Matrix in Loss of Muscle Force With Age and Unloading Using Magnetic Resonance Imaging, Biochemical Analysis, and Computational Models

**DOI:** 10.3389/fphys.2020.00626

**Published:** 2020-06-18

**Authors:** Usha Sinha, Vadim Malis, Jiun-Shyan Chen, Robert Csapo, Ryuta Kinugasa, Marco Vincenzo Narici, Shantanu Sinha

**Affiliations:** ^1^Department of Physics, San Diego State University, San Diego, CA, United States; ^2^Department of Physics, University of California, San Diego, San Diego, CA, United States; ^3^Department of Structural Engineering, University of California, San Diego, San Diego, CA, United States; ^4^Research Unit for Orthopaediic Sports Medicine and Injury Prevention, ISAG, Private University for Health Sciences, Medical Informatics and Technology, Hall in Tirol, Austria; ^5^Department of Human Sciences, Kanagawa University, Yokohama, Japan; ^6^Computational Engineering Applications Unit, Advanced Center for Computing and Communication, RIKEN, Saitama, Japan; ^7^Institute of Physiology, Department of Biomedical Sciences, University of Padua, Padua, Italy; ^8^Department of Radiology, University of California, San Diego, San Diego, CA, United States

**Keywords:** extracellular matrix, age and disuse related muscle force loss, structural muscle MRI, strain imaging, lateral transmission of force

## Abstract

The focus of this review is the application of advanced MRI to study the effect of aging and disuse related remodeling of the extracellular matrix (ECM) on force transmission in the human musculoskeletal system. Structural MRI includes (i) ultra-low echo times (UTE) maps to visualize and quantify the connective tissue, (ii) diffusion tensor imaging (DTI) modeling to estimate changes in muscle and ECM microstructure, and (iii) magnetization transfer contrast imaging to quantify the macromolecular fraction in muscle. Functional MRI includes dynamic acquisitions during contraction cycles enabling computation of the strain tensor to monitor muscle deformation. Further, shear strain extracted from the strain tensor may be a potential surrogate marker of lateral transmission of force. Biochemical and histological analysis of muscle biopsy samples can provide “gold-standard” validation of some of the MR findings. The review summarizes biochemical studies of ECM adaptations with age and with disuse. A brief summary of animal models is included as they provide experimental confirmation of longitudinal and lateral force transmission pathways. Computational muscle models enable exploration of force generation and force pathways and elucidate the link between structural adaptations and functional consequences. MR image findings integrated in a computational model can explain and predict subject specific functional changes to structural adaptations. Future work includes development and validation of MRI biomarkers using biochemical analysis of muscle tissue as a reference standard and potential translation of the imaging markers to the clinic to noninvasively monitor musculoskeletal disease conditions and changes consequent to rehabilitative interventions.

## Introduction

The loss of muscle mass with aging and with unloading (e.g., disuse) has been studied extensively. Accompanying the loss of muscle mass (termed sarcopenia) is a disproportionately greater loss of muscle strength as we age, termed dynapenia (Goodpaster et al., 2006). The role of muscular and neural determinants of muscle force loss with age has been investigated in several human and animal studies (Morse et al., 2005; Faulkner et al., 2007; Degens et al., 2009; Aagaard et al., 2010; Ballak et al., 2014). In addition to these determinants, the role of the extracellular matrix (ECM) in muscle force loss is also being increasingly recognized (Ramaswamy et al., 2011; Zhang and Gao, 2014).

The ECM of the muscle is arranged hierarchically with the endomysium around the muscle fiber, the perimysium surrounding a group of muscle fibers, and the epimysium around the whole muscle. Scanning electron microscopy (SEM) images of the connective tissue reveal that the endomysium is a highly ordered network that surrounds individual muscle fibers (Purslow and Trotter, 1994) while the perimysium includes extended cables organized as bundles of collagen fibers that terminates on muscle cells (Gillies and Lieber, 2011). Collagen is the main structural protein in ECM and may be present up to 10% by weight. Older muscles typically demonstrate fibrotic morphology (Lieber and Ward, 2013) that is characterized by an increase in collagen content and non-enzymatic cross-linking of collagen fibers (Haus et al., 2007), and results in a stiffening of the ECM (Wood et al., 2014). Measurements of the passive mechanical properties of the ECM reveal that it is inherently stiffer than muscle fibers and that the aging ECM has a higher modulus than the young ECM; the latter was attributed to an increase of densely packed extensively cross-linked collagen (Meyer and Lieber, 2011). Mechanical properties are measured on dissected, skinned single fibers placed in an activating solution using a device to measure force and length (Wood et al., 2014).

Aging and unloading significantly alter the concentration of force transfer proteins (e.g., decrease in dystrophin protein with aging) that transmit force both inside the muscle fibers (Hughes et al., 2017) and in the ECM (Kragstrup et al., 2011), changing their content, orientation, and composition. Age-related ECM remodeling process is accompanied by changes in the biochemical composition of the ECM, which features a higher ratio of type I to type III collagen fibers in older individuals (Hindle et al., 2009). In addition, augmented concentrations of type IV collagens but lower laminin contents have been observed in the basal lamina of slow twitch muscles of rat soleus and rectus femoris muscles (Kovanen et al., 1988). Transcriptional profiling studies performed in rat soleus muscles have shown that a large proportion of genes exerting a biological role in the ECM and cell adhesion is decreased in older muscles (Pattison et al., 2003). This observation suggests that the age-associated fibrogenic processes are driven by a decreased degradation capacity, rather than by increased formation of collagenous structures. In particular, the activity of matrix metalloproteinases, i.e., enzymes responsible for collagen degradation were reported to decrease at older age in rat gastrocnemius, digitorum profundus, and soleus muscles (de Sousa Neto et al., 2018). The interplay between age-associated ECM remodeling and the development of fibrosis in mouse gastrocnemius muscle is the subject of current investigations (Stearns-Reider et al., 2017; Forcina et al., 2019). As compared to the increasing number of studies investigating age-associated ECM remodeling, scant evidence exists on changes promoted by prolonged disuse. Early studies performed by Karpakka et al. (1990, 1991) showed that immobilization of rats led to reduced hydroxylase activity and hydroxyproline (an amino acid constituting collagens) in the soleus and tibialis anterior muscles. More recent studies, similarly performed in rodents, suggest that a disuse-induced increase in the activity of matrix metalloproteinase 9 (gelatinase B) may be responsible for decreased expressions of collagens I and IV (Giannelli et al., 2005; Liu et al., 2010). Changes in collagen content (in rat gastrocnemius, soleus, and tibialis anterior as well as in human vastus lateralis muscles) in response to short-term immobilization are usually small (Savolainen et al., 1988; Haus et al., 2007), which may be explained by the relatively slow turnover rate of collagenous structures. Prolonged disuse, however, may cause a proteolytic imbalance that is responsible for the breakdown of basal lamina structures. Such ECM remodeling may lead to altered permeability and, ultimately, promote disuse atrophy. It is important to note that aging and disuse may induce changes in the structure and biochemical composition of the ECM, which are expected to be associated with impaired transmission of contractile force through lateral pathways [i.e., through shearing between adjacent muscle fibers across costameres and the network of intramuscular connective tissue (IMCT)] (Csapo et al., 2020).

The current accepted structural models of muscle tissue include muscle fibers embedded in a matrix of connective tissue ECM. Force generated in the muscle fiber is transmitted to the ECM at multiple focal adhesions and subsequently to the tendon *via* the ECM (Huijing, 2003; Purslow, 2010). Myofascial pathways of force have been observed at various levels including between adjacent fibers (Huijing, 1999), multi-tendon muscle (Zhang and Gao, 2014), and muscle synergists (Bernabei et al., 2015). In multi-tendon muscles, force pathways exist through the myotendinous junction as well as through the ECM of the muscle bellies. In the multi-tendon extensor digitorum longus (EDL), it was shown that tenotomy severing the myotendinous junction had a smaller effect on the measured force than myotomy (severing the ECM between muscle heads) (Zhang and Gao, 2014). This effect was more pronounced in younger than aging rats, implying that lateral transmission of force (LTF) is reduced in the aging muscle. Architectural gear ratio (AGR, defined as whole muscle velocity to muscle fiber velocity), hypothesized to arise from the interplay of contractile forces and connective tissue constraints, has been identified as one of the determinants of mechanical performance in pennate muscles (Brainerd and Azizi, 2005, Eng et al., 2018). Pennate muscles can achieve a gear ratio greater than one since they can shorten through a combination of fiber shortening and rotation. Further, the gearing is variable across contractions with muscles operating at high gear during low force contractions and at low gear during high force contractions (Eng et al., 2018). Variable gearing seen in younger cohorts is lost in the muscle of aging rats presumably from the disruption in the interaction between contractile and ECM tissue leading to the decline in muscle performance (Azizi et al., 2008; Holt et al., 2016; Dick and Wakeling, 2017). AGR has been determined from *in situ* studies with sonomicrometry crystals embedded along with fibers to measure fiber length changes (Azizi et al., 2008), and from ultrasound (US) and MRI on human subjects (Shin et al., 2009b; Dick and Wakeling, 2017). Studies also show that the restriction of radial expansion by connective tissues results in decreased muscle fiber shortening and generation of less mechanical work (Azizi et al., 2017). Further, a stiff aging ECM may limit muscle performance possibly from both a smaller longitudinal contraction as well as a decrease in myofascial force transmission pathways *via* LTF (Ramaswamy et al., 2011, Zhang and Gao, 2014). In young rodent muscles, at least 80% of force transmission occurs laterally through the ECM (Huijing et al., 1998), whereas in frail old muscle, LTF through the ECM is reduced ~60% (Ramaswamy et al., 2011; Zhang and Gao, 2014).

The above summary clearly highlights that most of the tools used to study ECM are invasive, limiting comprehensive investigations of the contribution of the ECM to dynapenia in humans. This review focuses on structural and functional MRI to map ECM adaptations and consequence on muscle performance in healthy, aging, and disused muscle. Subject-specific anatomical (muscle compartments and morphology), voxel level compositional (fat, connective tissue, and muscle fractions), and fiber morphology (pennation angle and fiber length) models using MRI data will more accurately predict force output as well as force transmission pathways. Modeling-derived force transmission pathways can be used to validate MR derived biomarkers currently hypothesized to be surrogate markers of lateral transmission of force. It should be noted that the proposed MR indices are novel and validation with biopsy analysis, animal models, or computational models are either ongoing or need to be performed in future studies.

## Structural Imaging of the Ecm

The distribution and content of the ECM in humans have so far proven challenging to investigate with *in vivo* imaging, since its physical properties (very low spin-spin relaxation time, *T*2) renders it “invisible” in routine MRI. Consequently, there are few imaging-based studies published to date that have reported quantitative data of the ECM/fibrosis within skeletal muscles. Imaging these short *T*2 tissues requires special pulse sequences with ultra-low echo times (UTEs).

### Ultra-Low TE Imaging of Short T2 Intra-Muscular Connective Tissue

UTE imaging has been implemented by several groups and primarily used to study cortical bone, tendons, and cartilage (Sinha et al., 2007; Du et al., 2007a,b). Very few studies have investigated UTEs for muscle connective tissue quantification (Csapo et al., 2014; Ugarte et al., 2017a). Since adipose tissue is also present intramuscularly and a voxel can contain fat, water, and connective tissue fractions, and as these fractions change with age, it is important to be able to quantify all three tissues in muscle and investigate their variations with age. Fat quantification is an area of intense research and several recent excellent reviews on skeletal muscle adipose quantification in normal and diseased muscle are available (Grimm et al., 2018; Hu et al., 2019; Marty and Carlier, 2019). The IDEAL-IQ imaging sequence that accounts for the multiple fat resonance peaks has been validated for accurate fat fraction (Eskreis-Winkler et al., 2018).

**Figure 1:**
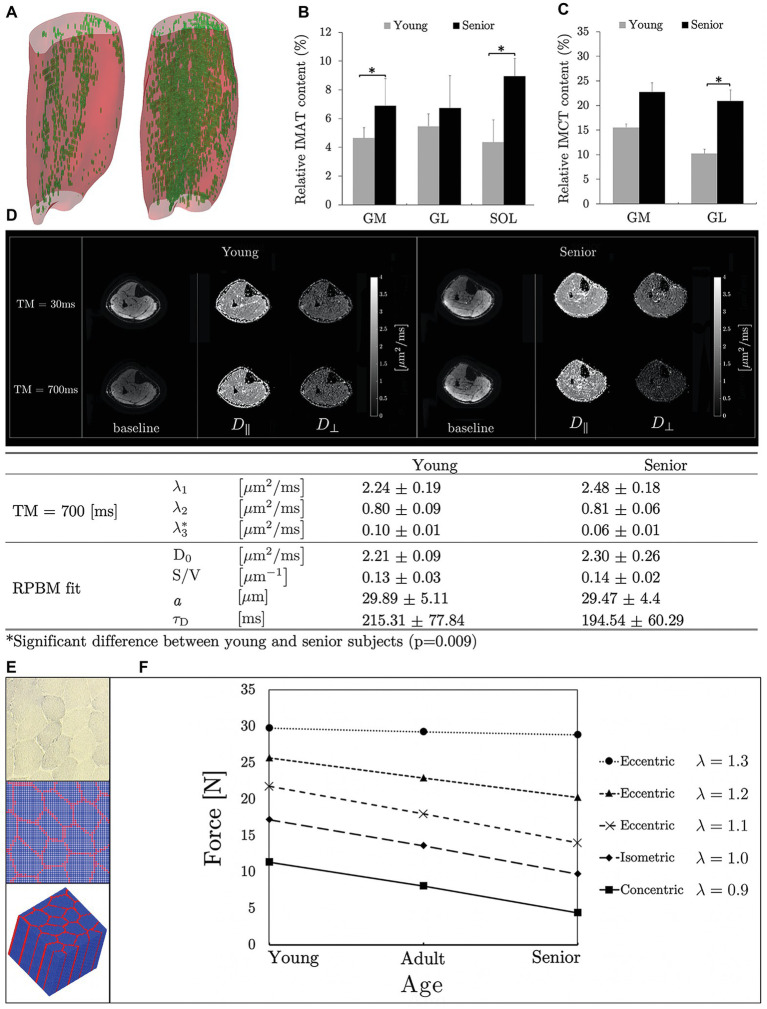
3D muscle volumes with the noncontractile tissue shown segmented (automated MCFCM algorithm) in green for a young (left) and aging (right) subject **(A)** reproduced with permission from Wiley publisher of Ugarte et al. (2017a). Box plots reproduced with permission from Springer publisher of Csapo et al. (2014) showing adipose **(B)** and connective tissue **(C)** content in young and aging subjects in the calf muscles (MG, medial gastrocnemius; LG, lateral gastrocnemius; SOL, soleus). Significant differences are marked by an asterisk. Baseline and eigenvalue (D_||_ and D_⊥_) images shown at two diffusion times (short: 30 ms and long: 700 ms) for young and aging subjects **(D)**. Tertiary eigenvalues decrease with diffusion time and are significantly different between young and aging subjects at longer diffusion times indicating that at these longer times when the average diffusion path length approaches fiber cell dimensions, the tertiary eigenvalue representing diffusion in the fiber cross-section is more sensitive to the fiber dimensions. The significant difference may potentially indicate that aging muscle fiber has a smaller diameter than young muscle fiber **(D)** reproduced with permission from ISMRM publisher of Malis et al. (2019a). Microstructural parameters of the model (*D*_0_, free diffusion coefficient; *S*/*V*, surface to volume ratio of fibers; *a*, muscle fiber diameter; and *τ_D_*, residence time, which is the average time spent within a muscle fiber) for young and aging subjects **(D)**. Histological image of muscle tissue (top), segmented cross-section (middle), and 3D model produced by extruding the 2D model along the *z*-direction (blue: muscle fiber, red: connective tissue) **(E)** reproduced with permission from Wiley publisher of Zhang et al. (2019). The micromechanical model prediction of force generated by three muscle types (young, adult, and old) is shown for isometric, concentric, and eccentric contractions. Young, adult, and old muscle tissue were modeled with passive stiffness as low, medium, and high and connective tissue thickness as thin, medium thick, and thick, respectively; *λ* is the stretch ratio **(F)** reproduced with permission from Wiley publisher of Zhang et al. (2019).

Intramuscular adipose tissue (IMAT) and IMCT in the triceps surae (TS) muscles have been quantified in a cohort of young and aging subjects, using quantitative fat/water and UTE sequences (Csapo et al., 2014). The latter study found an 18% greater TS muscle volume and a 39% higher muscle force in the younger cohort compared to the aging cohort (differences calculated from the average of young and aging subjects normalized to the average of all subjects), implying that 45% of force loss was not due to muscle size (ratio of volume to force differences). Further, in the gastrocnemius muscles (computed together for lateral and medial), IMCT increased by 64% and IMAT increased by 40% in the aging cohort, reflecting a 36% increase in the total amount of noncontractile tissue in the aging muscle (Figures 1A–C). A multichannel fuzzy cluster mean algorithm was implemented to automatically label IMAT/IMCT from UTEs images of calf images (Figure 1A; Ugarte et al., 2017a,b). Recent advances in image analysis enable accurate quantification of the CT, adipose, and water fraction in muscle from UTEs images (Araujo et al., 2017). US imaging also provides information on fat infiltration and fibrosis but is largely limited to 2D imaging and is semi-quantitative; however, US and MRI may provide complimentary information (Nijboer-Oosterveld et al., 2011; Arts et al., 2012; Mul et al., 2018).

### Magnetization Transfer Contrast Imaging

Quantification of the macromolecular fraction (such as collagen in connective tissue) can also be performed by quantitative magnetization transfer (qMT) imaging (Ramani et al., 2002). In the qMT method, macromolecules are selectively saturated with an off-resonance pulse and the effect of this saturation is seen as a decrease in signal intensity of the free protons. qMT models the signal intensity as a function of the magnetization transfer (MT) pulse offset and power to yield several parameters including the macromolecular fraction (Ramani et al., 2002). Recently, qMT has been applied to skeletal muscle and collagen has been proposed as the most likely macromolecule measured though it has not been validated with biopsy analysis (Sinclair et al., 2010; Li et al., 2014). A recent animal study to monitor muscle tissue formation found correlations between magnetization transfer ratio (MTR) with specific skeletal markers and to contractility; this study indicated the potential of MTR to serve as a surrogate marker of muscle fiber formation (rather than collagen) (Rottmar et al., 2016). Age-related changes in MT_sat_, a semi-quantitative index of MT showed that MT_sat_ decreases with age (White, 2019). The latter finding is surprising since if the measured macromolecule fraction reflects collagen, and collagen is known to increase with age, it would be anticipated that the MT_sat_ would increase with age (Alnaqeeb et al., 1984). These studies emphasize that validation based on biochemical analysis of biopsy samples is critical to identifying the macromolecules detected by qMT, MT_sat_, or UTEs techniques and is currently underway in the author’s laboratories.

### Diffusion Tensor Imaging and Modeling

Diffusion tensor imaging (DTI) is an established technique to probe the architecture of anisotropic structures such as skeletal and cardiac muscle fibers. Several excellent reviews discuss the imaging techniques, diffusion eigenvalues, and fractional anisotropy, and muscle fiber tracking including fiber length and pennation angles (Froeling et al., 2013; Oudeman et al., 2016). Diffusion eigenvalues reflect diffusion along the muscle fiber axis (primary eigenvalue) while secondary and tertiary eigenvalues maybe proportional to the fiber diameter (Galbán et al., 2004, 2005; Karampinos et al., 2007). However, the resolution of DTI-MRI precludes direct observations at the microscopic scale and modeling is required to link tissue microarchitecture to DTI indices.

Models for diffusion in muscle tissue were only proposed recently (Karampinos et al., 2009; Fieremans et al., 2017). A two-compartmental model, with elliptical muscle fibers as first compartment embedded in the second compartment, the ECM has been proposed (Karampinos et al., 2009). Model derived muscle fiber diameters of calf muscles (Karampinos et al., 2009) were in the range of muscle fiber diameters obtained from prior histological studies of excised muscle (Behan et al., 2002). Another model (Fieremans et al., 2017), termed as the random permeable barrier model (RPBM) fits the time dependence of D_⊥_ (average of the secondary and tertiary eigenvalues) to extract microstructural parameters. The time dependence of D_⊥_ is obtained from a stimulated echo sequence at diffusion times of the order of several hundred milliseconds (Figure 1D). Estimates of the fiber diameter in the calf muscle using RPBM were comparable to autopsy reports from literature (Polgar et al., 1973) though DTI modeling underestimated fiber diameters (Sigmund et al., 2014).

The two-compartment model applied to DTI data pre‐ and post-unloading showed that muscle fiber, permeability, and intracellular volume decreased and collagen increased while muscle ellipticity decreased post-suspension; the latter could potentially be explained by the lack of a mechanical stimulus during unloading (Malis et al., 2018b). The RPBM was applied to time dependent DTI data on old and young cohorts (Malis et al., 2019a). Significant differences were found in the tertiary eigenvalue at the longest diffusion times; the latter is most sensitive to the fiber diameter (Figure 1D). The RPBM model yielded a lower volume fraction for the aging muscle; the latter is a measure of the membrane’s ability to hinder diffusion. It is possible that aging muscle may have compromised sarcolemma integrity that makes it more permeable. While the DTI modeling with age and disuse are preliminary analysis, they confirm that modeling enables one to monitor fiber and ECM adaptations at the microstructural level (Malis et al., 2018b, 2019a).

## Strain and Strain Rate Mapping Using Dynamic Mri

Muscle tissue deformation is quantified using MRI sequences that map voxel displacement (displacement encoded imaging) or voxel velocity [velocity encoded phase contrast imaging, (VE-PC)] or MR tagging to track voxel displacements [spatial modulation of magnetization, (SPAMM)] (Tan et al., 2018). Strain heterogeneity along the muscle axis as well as the muscle fiber has been reported in the calf muscle and in the biceps brachii (Zhong et al., 2008; Shin et al., 2009b). Using the SPAMM technique, 3D strain and diffusion tensors were evaluated in the anterior tibialis and revealed a planar strain pattern where the principal shortening direction deviated from the muscle fiber direction indicating the presence of shear strain in the tissue (Englund et al., 2011). The deviation was attributed to the variations in fiber length leading to a spatial variability in the absolute length change that in turn results in a deviation of the average strain away from the fiber direction toward the longest fibers; the inter-fiber connections are mediated by the ECM (Englund et al., 2011). Heterogeneous length changes along the fascicles of human medial gastrocnemius muscle measured by combining magnetic resonance imaging deformation analyses and DTI were reported (Pamuk et al., 2016; Karakuzu et al., 2017). Computational models reveal that the strain heterogeneity arises primarily from a variation in fascicle lengths and the curvature of the fascicles. Nonuniform strain is hypothesized to affect force production and length range of force exertion (Yucesoy et al., 2002) and is an important determinant for the outcome of orthopedic surgery (Yucesoy et al., 2006; Yucesoy, 2010).

**Figure 2:**
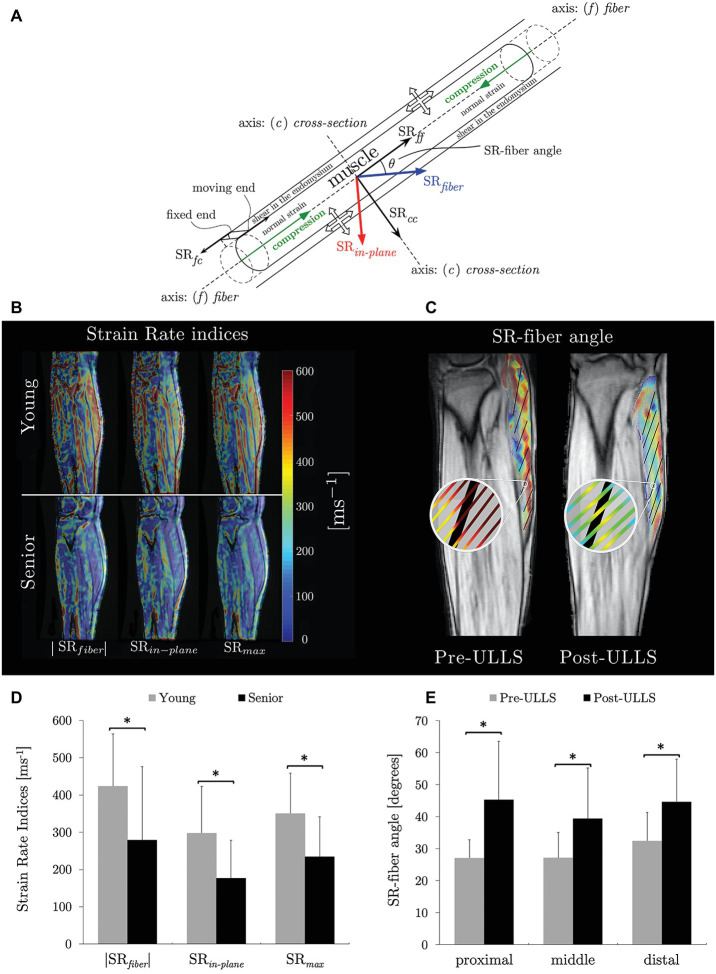
Schematic of a muscle fiber and endomysium with the principal basis and fiber basis defined with respect to the muscle fiber. The muscle fiber is shown contracting (green arrows) while the endomysium experiences a shear strain (end attached to the muscle contracts with the muscle fiber while the other end stays fixed resulting in a shear strain). The direction of the strain rate components in the two basis sets (SR*_fiber_* and SR*_in-plane_* in the principal basis and SR*_ff_* and SR*_cc_* in the muscle fiber basis) as well as the angle θbetween these two basis sets, SR-fiber angle are shown. The thick (unfilled) arrows show the lateral transmission of force pathways: from the cross-bridges of the myofilaments through the protein network on the sarcolemma to the extracellular matrix. Arrows indicate normal and shear strains **(A)** reproduced with permission from Wiley publisher of Sinha et al. (2018). Strain rate maps of the normal strain rates (SR*_fiber_* and SR*_in-plane_*) and shear strain rates (SR*_max_*) in a young and aging subject at the same force level. The color maps are color-coded according to the legend provided with the figure. Color maps are overlaid on the magnitude image at the corresponding frame; maps show lower values in both the normal strain rates in the aging cohort **(B)** reproduced with permission from Wiley publisher of Sinha et al. (2018). Streamlines of eigenvectors corresponding to SR*_fiber_* overlaid on fiber directions (in black, tracked from fascicle orientations) for one slice of the same subject pre‐ and post-suspension at the peak of the contraction phase. This shows the difference in angle enclosed by SR*_fiber_* and muscle fiber (SR-fiber angle) **(C)** reproduced with permission from Wiley publisher of Malis et al. (2018a). Box plots of SR indices evaluated in the MG that showed significant difference between young and aging subjects **(D)**. Box plots of SR-fiber angle showing significant differences in all three regions of the MG on unloading **(E)**. Significant differences in **(D)** and in **(E)** are denoted by ‘*’.

Recent studies focus on the extraction of strain rate (SR) indices from VE-PC images and the changes of these indices with age and with disuse (Sinha et al., 2015a, 2018; Malis et al., 2018a). The SR tensor was calculated from the spatial gradient of the velocity images (acquired during isometric contraction) and diagonalized to obtain the eigenvalues (SR*_fiber_* and SR*_in-plane_*) and eigenvectors. Figure 2A is a schematic of muscle fiber and ECM deformation during contraction specifying the SR components derived in the principal axis (SR*_fiber_* and SR*_in-plane_*) and in the muscle fiber axis (SR*_ff_* and SR*_cc_*) reference frames. Maximum shear strain (SR*_max_*) was calculated by rotating the SR tensor by 45° from the principal axis frame and the potential origin of the shear strain is schematically shown in Figure 2A (Sinha et al., 2015b). It was hypothesized that the MR measured shear strain arises from the shear of the endomysium as the muscle fiber contracts (Figure 2A). This hypothesis is in conformance with computational models (Gao et al., 2008a; Sharafi and Blemker, 2011). SR*_fiber_*, SR*_in-plane_*, and SR*_max_* were significantly lower in the aging cohort during isometric contraction (Figures 2B,D; Sinha et al., 2015a). On multiple variable regressions, maximum shear strain rate (SR*_max_*) and normal strain rate in the fiber cross-section (SR*_cc_*) were significantly associated with force (young and old cohorts) (Sinha et al., 2015a). In disuse atrophy, SR*_in-plane_* and SR*_fiber_* angle were significantly changed with unloading (Figures 2C,E). Changes in SR-fiber angle, SR*_in-plane_*, and shear SR as well as their ability to predict force and force changes may reflect the role of remodeling of the ECM in disuse atrophy and its functional consequence in reducing LTF (Malis et al., 2018a).

Computational models have identified that endomysium shear is the mechanism by which force is transmitted laterally (Gao et al., 2008b; Sharafi and Blemker, 2010). The experimentally measured MR shear strain was potentially attributed to the shear in the endomysium (Sinha et al., 2015a; Malis et al., 2018a). Further, shear SR was a significant predictor of force in the age and disuse studies highlighting that if shear strain is a measure of LTF, these results indicate the decreasing magnitude of LTF with age and with disuse (Sinha et al., 2015a, 2018; Malis et al., 2018a). The studies highlight that, while a direct noninvasive measurement of LTF is not possible, shear strain rate may potentially serve as a surrogate measure of LTF (Sinha et al., 2015a; Malis et al., 2018a). The increased stiffness of the aging muscle may result in lower shear in the ECM and this is confirmed in the experimental studies (Sinha et al., 2018). Recent advances in fast sequences integrating compressed sensing with VE-PC acquisitions have enabled the study of the full 3D strain tensor at different %MVC at significantly lesser number of contraction cycles (Malis et al., 2019b, 2020). It is anticipated that the ratio of shear to normal strains will decrease with increasing %MVC for aging subjects (compared to younger subjects) reflecting the decreased contribution of LTF in aging muscle.

US strain imaging based on speckle tracking has been used to measure strain and strain rate during skeletal muscle dynamic imaging as well as muscle stiffness (Lopata et al., 2010; Frich et al., 2019). High temporal resolution and real time capabilities are advantages while restriction to 2D-planes and limited field of view are disadvantages of US compared to dynamic MRI.

## Computational Modeling

Subject specific computational models using imaging data will more accurately predict force output as well as force transmission pathways (Blemker et al., 2007). MRI can provide anatomical (3D morphology of muscles including size and shape), voxel level compositional (fat, connective tissue, and muscle fractions), and fiber morphology (pennation angle, fiber length, and curvature) information enabling the generation of subject specific models.

The computational modeling of skeletal muscles has advanced from 1D lumped-parameter model (Bahler, 1968; McMahon, 1984; Zajac, 1989), 3D sophisticated macro-scale muscle models (Martins et al., 1998; Johansson et al., 2000; Lemos et al., 2004; Böl and Reese, 2008; Chi et al., 2010), to multi-scale models (Sharafi and Blemker, 2010; Virgilio et al., 2015; Spyrou et al., 2017, 2019; Zhang et al., 2019). Force transmission between the matrix and fibers using an interconnected fiber-matrix mesh discretization has been investigated (Yucesoy et al., 2002, 2006). Imaging advancements now allow measurements of the deformations of contracting skeletal muscles (Drace and Pelc, 1994; Maganaris et al., 1998; Pappas et al., 2002; Finni et al., 2003; Kinugasa et al., 2008; Shin et al., 2009a), and they also offer model parameters for computational investigation of deformation variations in muscle fibers and aponeuroses (van der Linden et al., 1998; Johansson et al., 2000; Yucesoy et al., 2002; Oomens et al., 2003; Blemker and Delp, 2005; Chi et al., 2010). Positive to negative strain variations in aponeuroses, nonuniform strain changes in sarcomeres, and asymmetrical muscle fiber cross-sectional deformations, have also been reported in a nonlinear FE muscle model (Chi et al., 2010). Several specially designed FE models have demonstrated how muscle force output can be strongly influenced by passive material properties (Chi et al., 2012). The reproducing kernel particle method (RKPM) (Chen et al., 1996, 2002) allows the model to be constructed directly from MR image voxels avoiding the complexity of mesh-based models and has been applied to muscle modeling (Basava et al., 2014).

A micromechanical model based on the concept of repeating unit cells extracted from histological cross-sections of rabbit muscles was proposed to study the effects of microstructural geometry in both fiber and fascicle levels on the macroscopic along-fiber shear modulus of skeletal muscle in normal and disease conditions (Sharafi and Blemker, 2010; Virgilio et al., 2015). Recently, a micromechanical computational model (Figure 1E) demonstrated that the increased stiffness and thickness of the passive materials of aging muscle tissue reduces the force generation capability under concentric contraction while maintaining the force generation capability under eccentric contraction (Figure 1F; Zhang et al., 2019).

While it has been acknowledged that the wealth of data from MRI can be leveraged to create subject specific models (Blemker et al., 2007), this has not been fully exploited. Subject specific models created for MRI data (including fiber direction from DTI) on a young and old subject predicted a force decrease with age that matched the experimental data (Basava et al., 2014). Further, the availability of functional MRI (strain patterns) provides experimental data for validation and fine tuning of computational models. It is also anticipated that subject specific endomysial thickness (MRI diffusion modeling) and stiffness (dynamic MRI) can be incorporated into micromechanical models (Zhang et al., 2019).

## Summary and Future Prospects

Advanced MRI techniques provide a number of indices to probe the structure and function of muscle fiber and the ECM. In order to establish these indices as imaging markers, MRI derived structural/functional indices await validation from biopsy studies. Structural MRI data can be used to generate subject specific computational models allowing predictions of force and strain distributions; the models in turn can be validated using the functional MR data. Future simulation studies using computational models may provide the causal link between shear in the ECM, shear strain from MRI, and lateral force transmission pathways allowing one to establish shear strain from MRI as a surrogate marker of LTF. In addition, a direct way to establish the link between *in vivo* MR strain indices and LTF will require animal model studies. It is acknowledged that MRI is only one of many tools including US imaging and biopsy analysis in the arsenal of tools for the comprehensive assessment of the ECM. The focus is to identify and validate MRI biomarkers, which then can be combined with markers from complementary modalities to advance the diagnosis, tracking of natural progression, and response to therapy in different musculoskeletal conditions ranging from sarcopenia, disuse atrophy to dystrophies.

## Author Contributions

US was the primary writer of the draft. VM, J-SC, RC, RK, MN, and SS contributed to sub-sections of the draft. US, VM, and SS contributed to the editing and proof-reading. VM produced the figures.

## Conflict of Interest

The authors declare that the research was conducted in the absence of any commercial or financial relationships that could be construed as a potential conflict of interest.
